# Molecular communication of the membrane insertase YidC with translocase SecYEG affects client proteins

**DOI:** 10.1038/s41598-021-83224-x

**Published:** 2021-02-16

**Authors:** Anja Steudle, Dirk Spann, Eva Pross, Sri Karthika Shanmugam, Ross E. Dalbey, Andreas Kuhn

**Affiliations:** 1 Institute of Biology, University of Hohenheim, 70599 Stuttgart, Germany; 2 Department of Chemistry and Biochemistry, Ohio State University, Columbus, OH 43210 USA

**Keywords:** Biochemistry, Biophysics, Microbiology, Molecular biology

## Abstract

The membrane insertase YidC inserts newly synthesized proteins by its hydrophobic slide consisting of the two transmembrane (TM) segments TM3 and TM5. Mutations in this part of the protein affect the insertion of the client proteins. We show here that a quintuple mutation, termed YidC-5S, inhibits the insertion of the subunit a of the FoF1 ATP synthase but has no effect on the insertion of the Sec-independent M13 procoat protein and the C-tail protein SciP. Further investigations show that the interaction of YidC-5S with SecY is inhibited. The purified and fluorescently labeled YidC-5S did not approach SecYEG when both were co-reconstituted in proteoliposomes in contrast to the co-reconstituted YidC wild type. These results suggest that TM3 and TM5 are involved in the formation of a common YidC-SecYEG complex that is required for the insertion of Sec/YidC-dependent client proteins.

## Introduction

Most bacterial membrane proteins are membrane-inserted during their biosynthesis by the Sec translocase and the YidC insertase^1^. Under certain conditions, the Sec translocase can form a large complex consisting of SecYEG, SecDF, YajC and YidC^2^. This Sec holo-translocon can also include the SecA protein that participates peripherally at the cytoplasmic face of the translocase complex using ATP hydrolysis to promote the movement of large periplasmic substrate domains^3^.

The main component of the translocase is the 10-spanning SecY protein that has an hour glass-like structure encompassing a hydrophobic ring at its center and an outer plug that controls the passage of a protein chain through the membrane^4^. Membrane proteins are released from SecY through its lateral gate consisting of TM2b, TM3, TM7 and TM8^5,6^. The lateral gate may be already involved in accepting a client protein’s hydrophobic segment as it emerges from the ribosome to enter the membrane. A ribosome arrested protein chain acting as an early insertion intermediate showed an interaction with the lateral gate by cryo-electron microscopy^7^.

The membrane insertase YidC of *E. coli* is a 6-spanning membrane protein that assists SecY for inserting membrane proteins and has been located next to its lateral gate in the holo-translocon structure^2^. In addition, YidC is capable of catalyzing membrane insertion of small proteins on its own, e.g. the coat proteins of the filamentous phage M13 and Pf3^8–10^. In this YidC-only pathway, the client protein enters by the hydrophobic slide generated by TM3 and TM5^11^. In contrast to the Sec translocase, YidC does not possess a transmembrane channel. Instead, it has a hydrophilic groove in the inner leaflet of the bilayer^12–14^ that may transiently harbour the periplasmic part of the client proteins prior to its translocation into the periplasmic space^15^.

An unresolved question is how YidC switches between a YidC-only mechanism and a SecYEG associated mechanism. Quantitation in *E. coli* cells had suggested that YidC is about tenfold more abundant than SecYEG^16^ and therefore exists in the two states, probably in a dynamic equilibrium. However, crosslinking of SecYEG and YidC has shown that their interaction is weak and multiple SecYEG crosslinks were observed at many positions around the YidC globule^17^. To determine the specific interaction between SecYEG and YidC the proteins were purified and their binding was measured in detergent solution and in reconstituted proteoliposomes using fluorescence resonance energy transfer (FRET). We show here that the SecYEG/YidC binding in detergent is saturated at a 1:1 ratio. Also, in proteoliposomes efficient energy transfer was observed when YidC and SecYEG were co-reconstituted. Moreover, a mutant YidC which is impaired in Sec-dependent insertion was deficient for SecYEG interaction and also no FRET occurred in the co-reconstituted proteoliposomes.

## Results

### A YidC mutant defective for Sec-dependent membrane insertion

**Figure 1:**
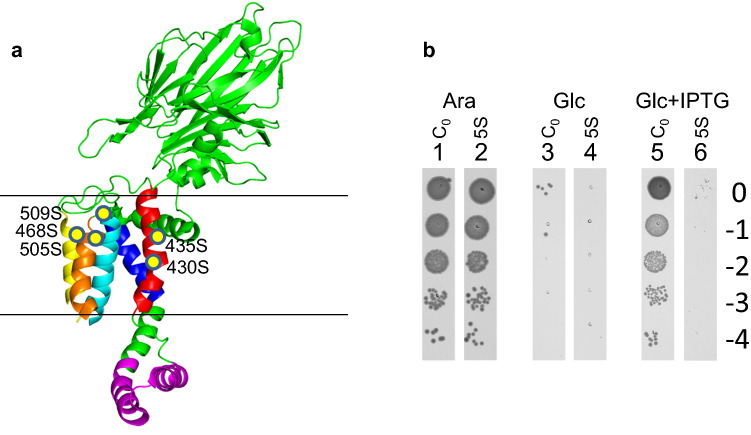
Construction and activity of a quintuple mutant YidC-5S. (**a**) Structure and topology of YidC-5S (PDB 3WVF). The locations of the 5 serine mutations are highlighted with yellow dots and are all located in the upper part of the hydrophobic slide of YidC; TM3 is coloured in red, TM4 in yellow and TM5 in gold. (**b**) Colony growth of MK6 cells with plasmids encoding the cysteine-less YidC_0_ (lane 1, 3, 5) or the YidC-5S mutant (lane 2, 4, 6). The cells were either grown on plates containing 0.2% arabinose that allow expression of the chromosomal *yidC* (lanes 1, 2), or on plates containing 0.4% glucose that repress the chromosomal *yidC* (lanes 3, 4), or on plates with glucose and 1 mM IPTG that induce the plasmid-encoded *yidC* (lanes 5, 6). Serial dilutions (tenfold, indicated on right margin) of the respective cultures were applied onto the agar plates and incubated at 37 °C overnight.

Since the hydrophobic slide of YidC constitutes the major substrate contact site, we had systematically mutated the involved residues located in the transmembrane segments TM3, TM4 and TM5 of YidC (Fig. 1a),^18^. A quintuple serine mutant, termed 5S with mutations at the residues 430, 435, 468, 505 and 509 was tested in the *E. coli* strain MK6 that has an arabinose inducible promoter upstream of the chromosomal *yidC* gene^18^. Depletion of the chromosomally encoded YidC was performed by plating the culture on glucose medium that resulted in cell death (Fig. 1b, lane 3). Whereas the induction of the plasmid-controlled cysteine-less wild-type C_0_ (lane 5) with IPTG allowed colony formation, the 5S mutant showed a growth defect and no colony growth was evident (lane 6).

**Figure 2:**
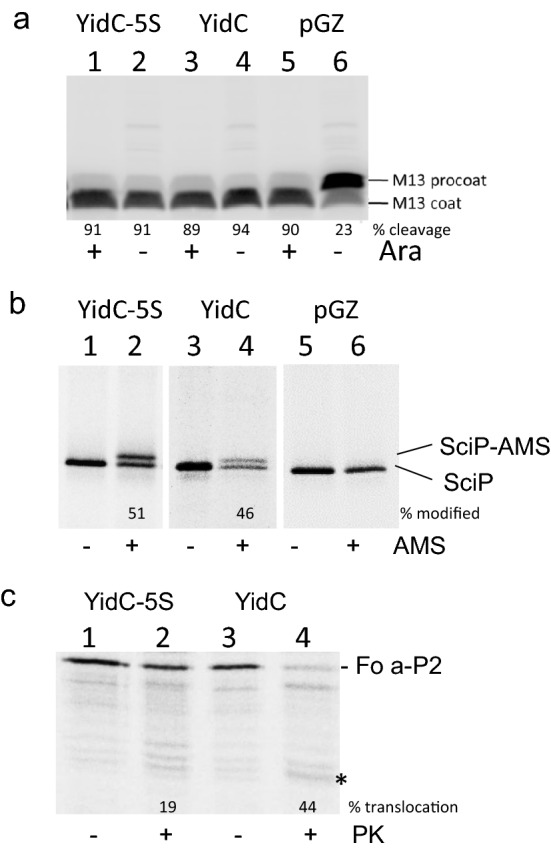
YidC-5S is fully functional for the membrane insertion of only Sec-independent substrates. The plasmid encoded YidC-5S mutant was tested for its function as a membrane insertase in *E. coli* MK6 under YidC-depleted conditions. A second plasmid encoding the substrate protein was co-transformed. The cells bearing the respective plasmids were grown in the presence of glucose to deplete the chromosomal YidC. As control, a culture bearing the vector (pGZ) was analysed in parallel (lanes 5, 6). (**a**) The expression of M13 procoat was induced with IPTG for 10 min and ^35^S-methionine was added for 1 min and the cells were analysed for cleavage of the M13 procoat protein to the mature coat in cells co-expressing YidC-5S (lane 2), YidC (lane 4) or with an empty plasmid (lane 6). For a control, the cleavage was followed under non-depleted conditions (+Ara, odd lanes). (**b**) SciP with a cysteine residue at 218 in the C-tail was co-expressed with YidC-5S (lanes 1, 2), YidC (lanes 3, 4) or where the empty plasmid was present (lanes 5, 6) and pulse-labelled for 2 min. The cells were non-treated (lanes 1, 3, 5) or treated with AMS to modify the cysteine and shift the protein by 0.5 kDa when the C-terminal tail was translocated to the periplasm (lane 2, 4, 6). (**c**) Membrane insertion of ATP synthase subunit a that was extended with the P2 domain of leader peptidase at the C-terminus was monitored in the YidC-depleted JS7131 cells. The cells expressing YidC-5S (lanes 1, 2) or YidC (lanes 3, 4) with subunit a-P2 were pulse-labelled with ^35^S-methionine for 1 min, converted to spheroplasts and analysed by protease mapping. Proteinase K was added (lanes 2, 4) or not (lanes 1, 3). When the protein was digested with Proteinase K, a protease-resistant fragment was generated (see asterisk). For a control, the cellular protection of cytosolic proOmpA and the digestion of OmpA by the protease were documented (Fig. S2). In all cases, the total cell protein was immunoprecipitated and analyzed by SDS-PAGE and phosphorimaging. The percentage of the cleavage of M13 procoat, of the modification of SciP with AMS and the protease accessibility of Fo-a-P2 were quantified and the values are indicated.

The 5S mutant was then tested for the insertion of various substrates. Surprisingly, the insertion of the YidC-dependent M13 procoat was not affected and the cleavage to coat was as efficient as was observed with the wild-type YidC (Fig. 2a). M13 procoat was pulse-labelled for 1 min with ^35^S-methionine and analysed by polyacrylamide gel electrophoresis (PAGE) after immunoprecipitation with an antiserum to coat. Only when the empty plasmid was present in the depleted cells, used as a control (lane 6; Fig. S1), did we observe an accumulation of the uncleaved procoat. Similarly, the YidC-5S mutant facilitated the translocation of the C-terminus of the C-tail protein SciP, a C-tail protein. SciP that has an additional cysteine residue at the translocated C-terminus^19^ was modified by 4-acetoamido-4′maleimidylstilbene-2-2′disodium sulfonate (AMS) added to the periplasm (Fig. 2b). Previously, it was shown that SciP uses YidC for its membrane insertion but is independent of the Sec translocase^19,20^.

In contrast, the Sec/YidC-dependent subunit a of the FoF1 ATP synthase was affected for membrane insertion in the YidC depleted cells when the 5S mutant was expressed (Fig. 2c). Previously it has been shown that the membrane insertion of subunit a with a C-terminal extension of the periplasmic region (P2) of leader peptidase requires YidC and SecYEG^21^. The plasmid-encoded protein was expressed in JS7131 cells and pulse-labelled for 60 s. Then the cells were converted to spheroplasts and Proteinase K was added to the periplasmic space of the spheroplasts to probe for the translocation of subunit a (Fo-a-P2). When wild-type YidC was expressed, Fo-a-P2 was digested and a protease-resistant fragment was generated (indicated with an asterisk in Fig. 2c, lane 4), whereas in the YidC-5S expressing cells Fo-a-P2 was mostly resistant to the proteinase indicating that the membrane insertion of Fo-a-P2 was affected. We noticed that the expression of Fo-a-P2 leads to a retardation of the processing of proOmpA (Fig. S2) which is possibly due to a stalling effect of the non-cleared substrate from the translocase. Such a stalling has been previously observed for Sec/YidC-dependent substrates when the YidC function was compromised^8,21^. Taken together, the 5S mutant that showed a growth defect in the complementation assay was functional for the insertion of the Sec-independent proteins M13 procoat and SciP but was defective in the Sec-dependent subunit a insertion. Possibly, the 5S mutations inhibit the YidC-SecYEG contacts that are required for the interaction of YidC with SecY which is required for the membrane insertion of subunit a.

### Purification and interaction of the fluorescently labelled YidC and SecYEG proteins

**Figure 3:**
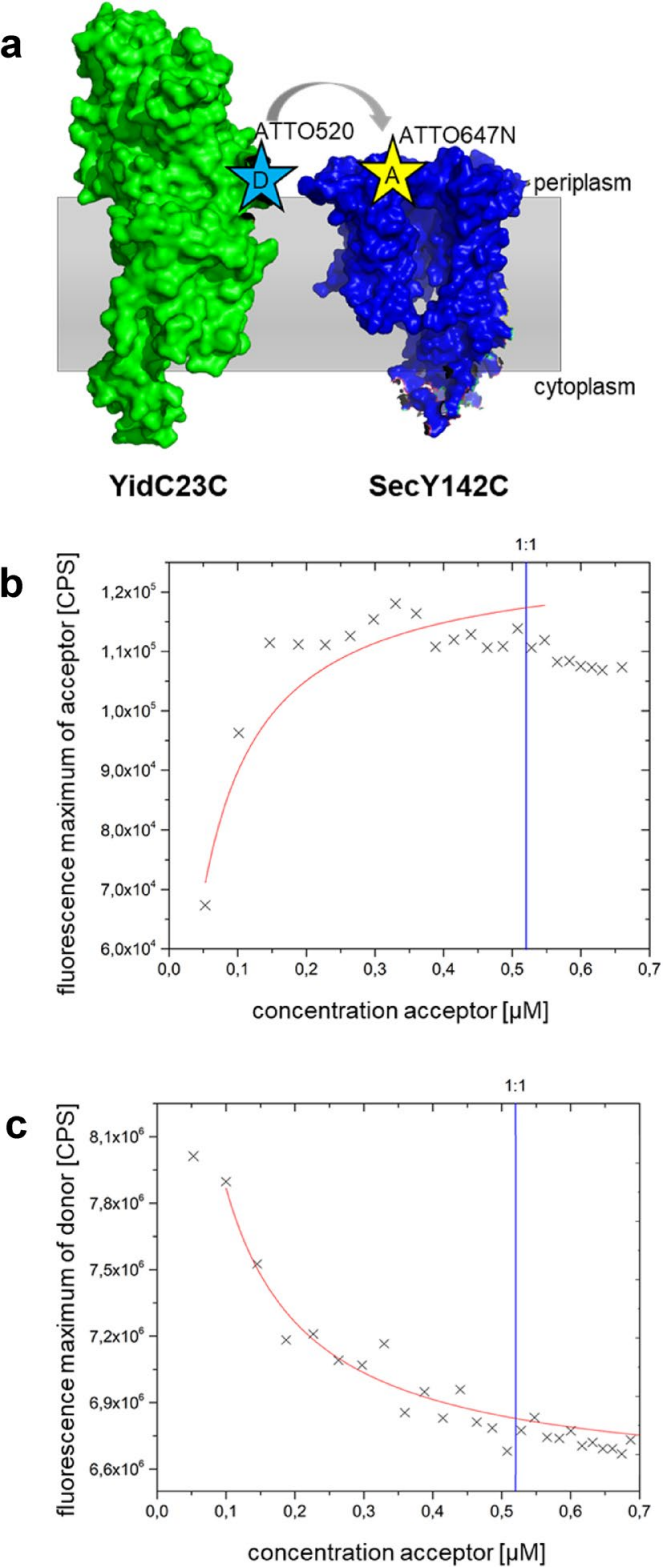
Fluorescence energy transfer (FRET) between YidC and SecYEG in detergent. (**a**) Schematic of the membrane topology of YidC (green; PDB 3WVF) and SecYEG (blue; PDB 3J01) and the positions that were labelled with Atto520 (at the cysteine residue 23 of YidC23C) and with Atto647N (at the cysteine 142 of SecY142C), respectively. Both proteins were purified prior labelling (see Supplementary Fig. S3). Fluorescence energy transfer was measured with the purified proteins in 0.03% DDM (**b**,**c**) The Atto520 labelled YidC was mixed with increasing amounts of Atto647N labelled SecYEG. The donor was excited at 516 nm and the fluorescence was followed between 520 – 750 nm. (**b**) The maxima of the acceptor fluorescence generated by FRET were monitored at 669 nm and the increase depended on the acceptor concentration. (**c**) The maxima of the donor fluorescence were monitored at 538 nm and decreased concomitantly with the increasing acceptor concentration.

To test whether YidC or YidC-5S can interact with SecYEG, a single cysteine residue was introduced into the proteins to allow site-specific fluorescent labelling. In YidC and YidC-5S, residue W23 was replaced by a cysteine (Fig. 3a). This residue is located at the periplasmic face of TM1, is well exposed and the substitution to cysteine does not affect its function^22^. In SecY, M142 in the periplasmic loop P2 was used to introduce a single cysteine that is well-exposed in the periplasmic space^23^. The purified YidC protein was labelled with Atto520 and SecYEG with Atto647N (Figs. S3 and S4) with an efficiency of 18% and 24%, respectively. For the binding experiments, we used 0.55 µM YidC in 0.03% dodecyl maltoside and titrated increasing amounts of SecYEG. The donor fluorescence of YidC in the sample was excited at 516 nm. When the fluorescence of acceptor was recorded at 669 nm, energy transfer was observed and increased with the increasing acceptor concentration (Fig. 3b). A maximum of the FRET signal was reached when SecYEG attained a molar 1:1 ratio with YidC. Simultaneously, the donor fluorescence decreased which was recorded at 538 nm (Fig. 3c). The binding affinity between YidC and SecYEG in detergent was determined with a K_d_ of 27 (± 10) nM (Fig. S5). It is important to note that in these ensemble FRET experiments only a portion of the donor fluorescence energy is transferred to the acceptor and a variety of distances may exist between the dyes and the signal results from an average of these distances.

### Reconstitution of YidC and SecYEG into proteoliposomes

**Figure 4:**
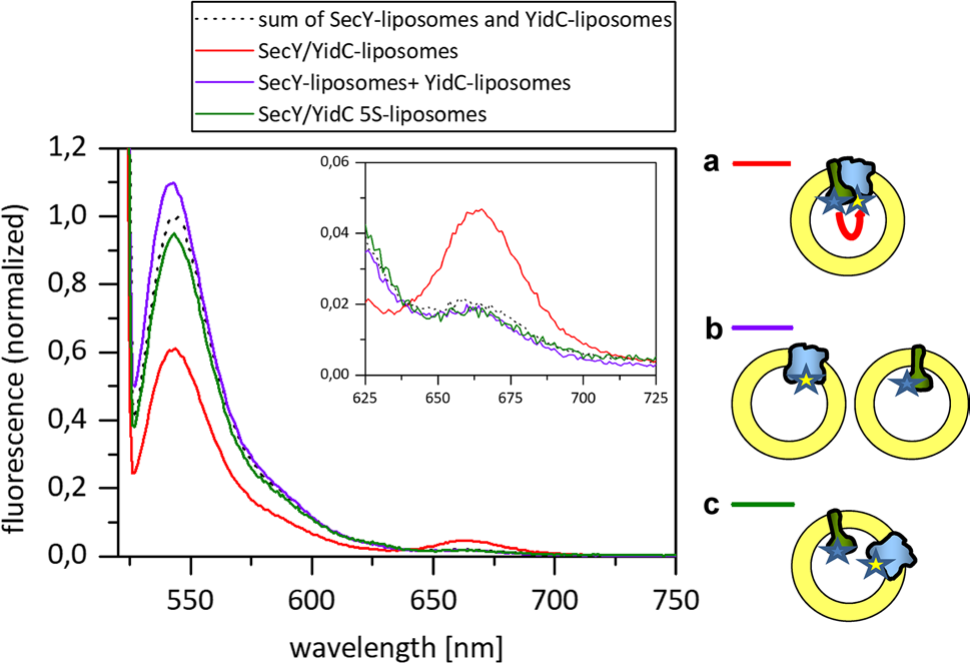
Fluorescence energy transfer (FRET) between YidC and SecYEG in proteoliposomes. Both YidC-Atto520 and SecYEG-Atto647N were reconstituted into proteoliposomes. (**a**) When both proteins were reconstituted simultaneously and were present in the same proteoliposome FRET was observed, resulting in a decrease of the donor fluorescence (red line) and increase of the acceptor fluorescence when compared with the sum of the fluorescence with the single proteins (black dotted line). (**b**) No FRET was observed when YidC and SecYEG were reconstituted into separate proteoliposomes and mixed (purple line). (**c**) As in a, but Atto520 labelled YidC-5S was combined with SecYEG-Atto647N in the same proteoliposomes (green line). The reconstitution of the proteins is shown in Fig. S4.

The purified and fluorescently labelled proteins were separately reconstituted with 1,2 dioleoyl-sn-glycero-3-phosphocholine (DOPC) into proteoliposomes. For the reconstitution a molar protein-lipid ratio of 1:1000 was chosen. Equal amounts of the reconstituted proteoliposomes were mixed and the fluorescence was recorded for the YidC-proteoliposomes, the SecYEG-proteoliposomes and the combined proteoliposomes (Fig. 4). The signal of the combined proteoliposomes (purple line) was compared to the sum of the two separate proteoliposomes (black dotted line). The match of the two curves shows that no FRET was detectable. In contrast, when YidC and SecYEG were co-reconstituted (red line) the donor signal (at 538 nm) decreased and the acceptor signal increased (at 669 nm) when compared to the signal of the sum of the two separate proteoliposomes (black dotted line). A clear increase of the acceptor signal concomitant with a decrease of the donor signal was observed when SecYEG and YidC were present in the same liposome (a). This was not the case when SecYEG and YidC were present in separate liposomes (b).

The Atto520 labelled YidC-5S mutant was then co-reconstituted with SecYEG and analysed in the same way (green line; Fig. 4c). Only small differences to the control were detectable (black dotted line). These results show that the YidC-5S mutant is impaired for SecYEG interaction when present in the same proteoliposome. In a titration experiment with detergent-solubilized YidC-5S and SecYEG only little, non-specific binding was observed (Fig. S6).

### Disulphide cross-linking points to the SecY and YidC interacting residues

**Figure 5:**
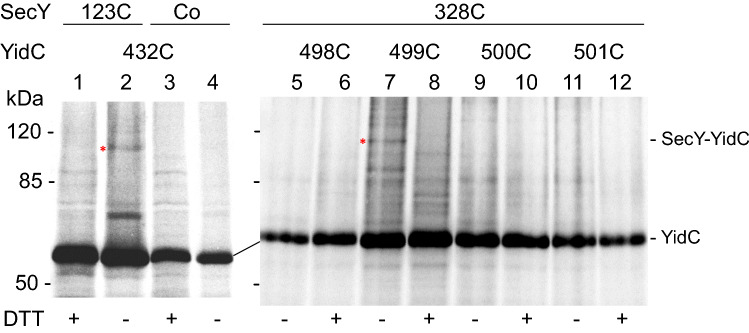
YidC and SecY show cross-links between the hydrophobic slide and the lateral gate. Single cysteine mutants of YidC and SecY were generated and co-expressed in *E. coli* MK6. YidC432C was coexpressed with SecYEG that had a single cysteine at position 123 in TM3 of SecY (lanes 1, 2). The cells were pulse-labelled with ^35^S-methionine and treated with 200 µM DTNB (all lanes) followed by 1 mM DTT (lanes 1, 3, 6, 8, 10, 12) in some cases and analysed by immunoprecipitation with an antiserum to YidC and phosphorimaging. For a control, SecYC_0_ was coexpressed with YidC432C (lanes 3, 4). SecY that has a single cysteine at residue 328 in TM8 was coexpressed with the YidC mutants that have a single cysteine either at 498, 499, 500 or 501 in TM5. A cross-link band was observed between SecY328C and YidC499C (red asterisk). The expression of SecY was monitored on a Western blot (Fig. S7).

Single cysteine mutants of SecY and YidC, respectively, were used to pinpoint the residues that are involved in a direct molecular interaction. The respective plasmids were co-transformed into *E. coli* MK6 cells, induced with 1 mM IPTG for 20 min, pulse-labelled with ^35^S-methionine for 3 min, treated with 200 µM DTNB and immunoprecipitated with an antiserum to YidC (Fig. 5). When a cysteine residue was placed at position 123 in the TM3 segment of SecY and at position 432 in the TM3 segment of YidC a cross-linked band was detected (lane 2) that did not appear in the presence of DTT (lane 1). Also, when a cysteine-less SecY variant was co-expressed with YidC 432C, no cross-linked band was detectable (lane 4) although the cellular amount of SecY was not affected (Fig. S7).

Likewise, single cysteine mutants showed that TM8 of SecY interacts with TM5 of YidC. When a cysteine residue was placed at position 328 of SecY (TM8) and 499 of YidC (TM5) a cross-linked band was detected (lane 7) that disappeared when the cells were treated with DTT (lane 8). Taken together, the disulphide cross-linking data show that the lateral gate of SecY, consisting of TM3 and TM8 directly interacts with the hydrophobic slide of YidC, namely the TM3 and TM5.

## Discussion

**Figure 6:**
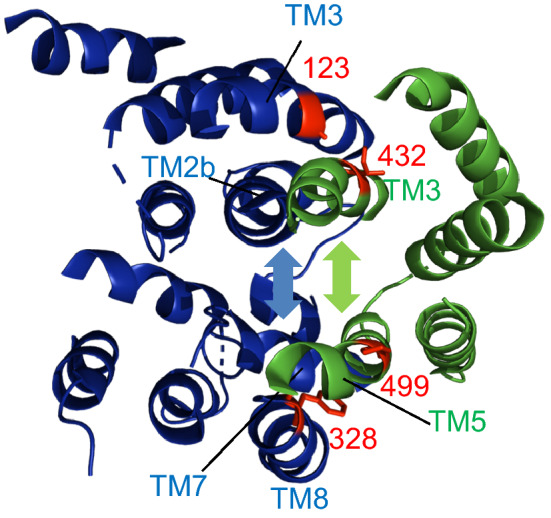
Model of a transmembrane complex between the lateral gate of SecY and the hydrophobic slide of YidC. The SecY TM1-10 (PDB 3J01) is coloured blue, and the YidC TM2-6 (PDB 3WVF) is coloured green. The residues of SecY (G123 and F328) contacting YidC (I432 and P499) are in red. The TM of the lateral gate (SecY-TM2b, 3, 7 and 8, blue arrowhead) interacts with the hydrophobic slide (YidC TM3 and 5, green arrowhead). Therefore, the pore of SecY and the hydrophilic groove of YidC could form an extended cavity for hosting a substrate.

For membrane insertion, a close interaction between the translocase SecYEG and the membrane insertase YidC is required for most of the membrane proteins that use the Sec/YidC dependent pathway. Previously, the existence of the “holo-translocon” has been demonstrated under overproducing conditions^2^. A low-resolution structure was presented that suggests that the lateral gate of SecY and the TM3 of YidC are in contact^23^. We present here further evidence that the lateral gate of the Sec translocase (TM3 and TM8) directly interacts with YidC (TM3 and TM5, respectively), by disulphide cross-links in vivo (Fig. 5). The close interactions between the lateral gate and the hydrophobic slide could generate an extended cavity between the pore of SecY and the hydrophilic groove of YidC in the complex (Fig. 6). This cavity could then be used to support the insertion of large membrane proteins allowing the substrate proteins to enter the YidC/SecYEG complex between the hydrophobic slide and the lateral gate. Indeed, a recent cryo-electron microscopic structure of a partially inserted proOmpA protein showed the involvement of TM3, TM7 and TM8 of the SecY lateral gate in the early insertion events^7^.

To further explore the interaction and the binding affinity between SecYEG and YidC we used fluorescence resonance energy transfer (FRET) in vitro. The two labelled and purified proteins interact in detergent with a high affinity, which, however, might not reflect the in vivo situation. To investigate the interaction in the membrane both proteins were reconstituted first in separate liposomes (Fig. 4b). In this case, no FRET between SecYEG and YidC was observed. However, co-reconstitution of SecYEG and YidC led to an efficient FRET signal (Fig. 4a) that corroborates an interaction of the two components. In cells, the interaction between SecYEG and YidC might be different and influenced by additional factors. Previously, it was shown that SecF is directly involved in contacting YidC residues^24,25^. Also, multiple positions between the Sec translocase and YidC were identified as weakly interacting residues^17^.

Further support for the functional interaction between the Sec translocase and the hydrophobic slide of YidC is that the YidC-5S mutant that is defective in SecYEG interaction is partially inhibited in membrane insertion of a YidC/Sec-dependent substrate (Fig. 2c). When five of the hydrophobic slide residues, namely M430, A435, P468, F505 and F509 were substituted with serines, the interaction to SecYEG was severely reduced (Fig. 4c). All these mutations are localized in the upper part of the hydrophobic slide close to the periplasmic leaflet of the membrane. The YidC-5S mutant is affected to insert subunit a of the ATP synthase into the membrane. This protein has been studied with a carboxyl-terminal extension of the leader peptidase P2 domain^21^. Whereas the translocation of the amino-terminal domain of subunit a required SRP, YidC and the membrane potential, the translocation of the central loop is dependent on SRP, the SecYEG translocase and YidC.

To our surprise, the five serine mutations in the hydrophobic slide had no negative effect on the insertion of the Sec-independent substrates M13 procoat and SciP (Fig. 2). Only when additional contacting residues were changed to serines, namely F424, L427, F476 and F502 in the YidC-9S mutant, was a Sec-independent substrate affected^11^. Presumably, the initial step to insert a YidC-only substrate is promoted by the hydrophobic residues close to the cytoplasm of the slide^15^.

We also found that YidC-5S cannot complement the depletion of wild-type YidC and is defective for growth (Fig. 1b). In conclusion, the Sec-dependent function of YidC is essential similar to the Sec-independent function of YidC as had been shown earlier^26^. Since the insertion of CyoA as an essential component of the ubiquinol oxidoreductase and the subunit c of the ATP synthase require YidC for insertion it was concluded that the depletion of YidC affecting Sec-independent substrates dissipates the transmembrane potential. This might be the main reason to cause cellular death.

Taken together, we show here that the YidC-5S mutant is defective in interacting with SecYEG and the insertion of Sec/YidC dependent subunit a of the ATP synthase into the membrane is affected. This can also explain why the mutant is not capable of complementing the YidC depletion strain and prevents normal cell growth.

## Methods

### Bacterial strains and plasmids

YidC depletion was carried out using *E. coli* strains MK6^11^ and JS7131^8^. The latter has a *yidC* gene with the *araC-araBAD* promoter in the lambda attachment site and a deletion in the original *yidC* gene. In the MK6 strain, the promoter of the *yidC* gene had been exchanged with the *araC-araBAD* promoter cassette. Depletion of the chromosomally encoded YidC was achieved by growth of MK6 in the presence of 0.4% glucose. For complementation assays, MK6 was transformed with pGZ119EH^27^ expressing the respective YidC proteins under the control of the *tac* promoter. Co-expression of M13 procoat was performed with pMS-M13 procoat^28^, SciP with pMS-SciP-C^29^ and pMS-Su-a-P2^21^, derivatives of the pMS119EH vector^30^. For the purification of SecYEG and YidC *E. coli* SF100 and C43 were used, respectively^5,29^.

### Translocation assay with the wild-type M13 procoat

The wild-type M13 procoat expressed from a pMS119EH derivative was co-expressed with YidC-5S or YidC to test for their capability of inserting a substrate. Overnight cultures of MK6 harboring two plasmids, one encoding the YidC mutants or the YidC control (in pGZ119EH) and the other the wild-type M13 procoat (in pMS119EH), were grown in LB (200 µg/mL ampicillin, 25 µg/mL chloramphenicol, 0.2% arabinose, 0.4% glucose) overnight at 37 °C. The cells were washed twice with LB medium and then inoculated 1:100 in fresh LB medium (200 µg/mL ampicillin, 25 µg/mL chloramphenicol, 0.4% glucose) and grown under YidC depletion conditions to an OD_600_ = 0.5 at 37 °C. 200 µL were taken, washed twice and resuspended in 200 µL M9 medium lacking methionine. After 45 min growth at 37 °C, 1 mM IPTG was added for 10 min. 20 µCi [^35^S]-methionine was added for 1 min, followed by the addition of 10% TCA. The samples were then processed for immunoprecipitation and PAGE as previously described^29^.

### Translocation assay of SciP with AMS

The pMS119EH plasmid-encoded SciP-C^19,29^ was co-expressed with pGZ119EH encoding YidC-5S in MK6 cells under YidC depletion conditions. The cells were grown in LB (100 µg/mL ampicillin, 37 µg/mL chloramphenicol, 0.4% glucose) to an OD_600_ = 0.5 at 37 °C. Then 1 mL culture was sedimented, washed and resuspended in M9 minimal medium lacking methionine and split into two cultures (+/− AMS). The cells were grown for another 45 min at 37 °C, induced with 1 mM IPTG for 10 min. 2.5 mM AMS (Molecular Probes) was added for 1 min followed by pulse labelling with 15 µCi ^35^S-methionine for 2 min. Nonradioactive l-methionine was added for 10 min, and the AMS reaction was quenched by the addition of 10 mM DTT for further 10 min. The cells were acid-precipitated and immunoprecipitated (his tag antibody) as previously described^29^.

### Membrane translocation of subunit a of ATP synthase

The ATP synthase subunit a-P2 was co-expressed with one of the YidC constructs to examine membrane insertion following the protocol of Spann et al.^29^. Overnight cultures of the YidC depletion strain JS7131 bearing two plasmids, one encoding the YidC defective mutant or the YidC blank control (pGZ119) and the other encoding subunit a-P2 (pMS119), were grown in LB (50 μg/mL ampicillin, 25 μg/mL chloramphenicol, 0.2% arabinose) overnight at 37 °C. After washing the cells twice with LB medium, the cells were inoculated 1:100 in fresh LB medium (50 µg/mL ampicillin, 25 µg/mL chloramphenicol, 0.2% glucose) and grown under YidC depletion conditions for 4 h. The cells were then washed with M9 medium and transferred into M9 lacking methionine, and grown for 30 min at 37 °C. Then, the culture was induced with 1 mM IPTG for 7 min, and then pulse-labelled with [^35^S]-methionine for 1 min. Cells were collected by pelleting in a microfuge at 4 °C and resuspended in 40% sucrose and 33 mM Tris–HCl (pH 8.0) and converted into spheroplasts by treatment with lysozyme (5 µg/mL) and 1 mM EDTA (pH 8.0) on ice for 30 min. The spheroplasts were treated with or without proteinase K (0.8 mg/mL final concentration) for 1 h on ice. 5 mM PMSF (final concentration) was added to stop the protease reaction. Subunit a-P2 was immunoprecipitated with leader peptidase antibody and the samples were analyzed by SDS-PAGE and phosphorimaging.

### In vivo YidC complementation assay

The *E. coli* MK6 strain with the chromosomal YidC under the control of the *araC-araBAD* promoter was transformed with a pGZ119EH plasmid carrying the *yidC-5S* or the *yidC*-*wildtype* gene as a positive control or with a vector without an insert as a negative control. Cultures were grown at 37 °C in LB medium supplemented with 25 µg/mL chloramphenicol and 0.4% glucose. After reaching an OD_600_ of 1.0 and at least 3 h for complete depletion of chromosomal YidC, serial dilutions were performed. The diluted cultures (2 µL each) were applied onto LB agar plates containing 25 µg/mL chloramphenicol and either 0.4% glucose for depletion or 0.2% arabinose as a control. For the plasmid-derived expression the agar plates contained 1 mM IPTG. The plates were incubated overnight at 37 °C.

### Purification of YidC and SecY

For purification, 2 L of LB medium containing 12.5 µg/mL chloramphenicol or 100 µg/mL ampicillin, respectively, was used to grow the *E. coli* C43 cells bearing pGZ119-YidC-5S or pMS119EH-YidC-wt or *E. coli* SF 100 cells bearing pTrc99a-SecYEG plasmid^31^. After reaching an OD_600_ of 0.6 for YidC and 0.9 for SecYEG, respectively, protein expression was induced for 2 h with 0.5 mM IPTG and the cells were harvested by centrifugation (7000 g, 4 °C, 10 min). The cells were shock-frozen in buffer 1 (20 mM Tris–HCl pH 7.5 for YidC and pH 8.0 for SecYEG, 300 mM NaCl, 10% glycerol) supplemented with 1 mM PMSF and 1 mM DTT. To the thawed cells 1 mM MgCl_2_ and DNase I were added. After cell disruption by a One-Shot cell disruption system (Constant Systems Ltd) and separating the cell debris by centrifugation (17,500 g, 4 °C, 20 min), the membranes were harvested by ultracentrifugation (140,000 g, 4 °C, 90 min) and resuspended in buffer 1. The membranes were solubilized by adding 1% DDM for YidC or 2% DDM for SecYEG and incubation for 2 h at 4 °C. After another ultracentrifugation step (95,000 g, 4 °C, 30 min), the supernatant was incubated for 1 h at 4 °C with 1 mL (YidC) or 0.5 mL (SecYEG) of equilibrated Ni–NTA and 20 mM (YidC) or 30 mM (SecYEG) imidazole in a total volume of 50 mL on a rotary wheel for immobilized metal ion affinity chromatography (IMAC). The column was run by gravity flow and the Ni–NTA was washed with 10 column volumes (CV) washing buffer (YidC: 20 mM Tris–HCl pH 7.5, 300 mM NaCl, 10% glycerol, 30 mM imidazole pH 7.5, 0.03% DDM; SecYEG: 20 mM Tris–HCl pH 8, 300 mM NaCl, 10% glycerol, 50 mM imidazole pH 8, 0.03% DDM). The protein was eluted with 10 CV elution buffer (YidC: 20 mM Tris–HCl pH 7.5, 300 mM NaCl, 10% glycerol, 300 mM imidazole pH 7.5, 0.03% DDM; SecYEG: 20 mM Tris–HCl pH 8, 300 mM NaCl, 10% glycerol, 300 mM imidazole pH 8, 0.03% DDM) in 1 mL fractions. 20 µL of each elution fraction were analyzed by SDS-PAGE and Coomassie staining. Elution fractions containing protein were shock-frozen and stored at − 80 °C.

### Fluorescence labelling of YidC and SecY

For labelling, the fluorescence maleimide dyes Atto520 for YidC and Atto647N for SecY were used. A 1.3-fold molar amount of dye in DMSO was added to the protein solution and incubated rotating for 30 min at room temperature and overnight at 4 °C. The reaction was then quenched by incubation of the solution with 10 mM DTT for 2 h at 4 °C. The labelled proteins were separated from the non-bound dye by size exclusion chromatography using a Superdex 200 10/300 at RT in elution buffer (see purification) without imidazole.The labelling efficiency of SecY was 24%, of YidC 18% and YidC5S 34%. The collected fractions were analyzed by SDS-PAGE (15%) and visualized by fluorescence detection and Coomassie staining (see Supplementary Fig. S3). The labelled proteins were shock-frozen and stored protected from light at − 80 °C.

### Reconstitution of YidC and SecYEG in proteoliposomes

YidC and SecYEG were reconstituted into DOPC-liposomes by the extruder and Bio-Bead method. The proteins were dialyzed to remove the glycerol, mixed in a molar lipid-to-protein ratio of 1:1000 with liposomes and extruded 15 times. To remove the detergent, the proteoliposomes were twice rotated with activated Bio-Beads for 1 h at 4 °C. The proteoliposomes were collected by centrifugation (Beckman Airfuge, 20 psi, 10 min, 4 °C), resuspended in buffer 1 (see purification) without glycerol and immediately used for measurements.

### Förster resonance energy transfer (FRET) measurements

For measuring FRET, the donor dye (Atto520) was excited at 516 nm (Fluorolog, HORIBA Jobin Yvon GmbH). The fluorescence spectrum was recorded at 520–700 nm to detect the fluorescence maximum of the donor at 538 nm and the fluorescence maximum of the acceptor at 669 nm.

### FRET data analysis

To determine the K_d_ value of the SecY-YidC interaction in detergent a titration was performed and the fluorescence spectrum of each titration point was recorded. Because of the increasing volume and the addition of acceptor, the concentration of the labelled proteins changed during titration. To correct this effect of dilution and concentration, respectively, buffer controls (buffer to YidC-Atto520 and SecY-Atto647N to buffer) were performed equivalent to the titration of the proteins. The corrected maxima of the signals were plotted against the concentration of the acceptor protein (SecY-Atto647N) and linearized according to Lineweaver–Burk to determine the K_d_ value. For the measurements on the reconstituted samples, no buffer controls were needed. To determine the background signal, the proteins were reconstituted separately, in addition to the mixed samples. The sum of the signals of the two separately measured SecYEG and YidC proteoliposomes was used for normalizing the signal of the mixed samples to determine the increase or decrease of the fluorescence because of FRET.

### In vivo disulphide cross-linking

For disulphide cross-linking, single cysteine mutants of SecY and YidC were prepared. The *E. coli* MK6 strain with the chromosomal YidC under the control of the *araC-araBAD* promoter was co-transformed with a pTrc99a plasmid carrying the mutated *secYEG* genes and a pGZ119EH plasmid carrying the mutated *yidC* gene. Cultures were grown at 37 °C in LB medium supplemented with 100 µg/mL ampicillin, 12.5 µg/mL chloramphenicol and 0.4% glucose. After reaching an OD_600_ of 0.4 to 0.5 following growth for 3 h for depletion of chromosomal YidC, 400 µL of the cultures were washed twice with M9 minimal medium and incubated in 400 µL M9 minimal medium for 45 min at 37 °C. After induction with 1 mM IPTG for 20 min, the proteins were pulse-labelled with ^35^S-methionine for 3 min and treated for 10 min with freshly prepared 200 µM DTNB for oxidation. The protein pellets after TCA precipitation were washed with acetone and boiled for 5 min at 95 °C in 100 µL 2% SDS/10 mM Tris-buffer (pH 8). 1 mL of cold TENTX-buffer (150 mM NaCl, 10 mM Tris, 1 mM EDTA, 2% Triton-X 100, pH 8) and 20 µL Staph A (10% in PBS) were added for reabsorption (45 min). For the immunoprecipitation, antiserum to YidC was added to the supernatant and incubated overnight. After precipitation with 30 µL Staph A and washing twice with TENTX-buffer and once with TENTX-buffer without Triton-X 100, the samples were divided in half. The samples were resuspended in SDS sample buffer with or without DTT and analyzed by SDS-PAGE and following phosphor imaging.

## Supplementary information

Supplementary information.

## Data Availability

The data that support the findings of this study are available from the corresponding author upon reasonable request including the raw data of Figs. 3, Fig. 4 and Fig. S6.
