# Supplementary material for: Molecular communication of the membrane insertase YidC with translocase SecYEG affects client proteins
Source: Sci Rep. 2021 Feb 16;11:3940. doi: 10.1038/s41598-021-83224-x (PMC7886851; doi:10.1038/s41598-021-83224-x)
Supplement: Supplementary file 1 — Supplementary information. [file 41598_2021_83224_MOESM1_ESM.docx]

Molecular communication of the membrane insertase YidC with translocase SecYEG affects client proteins

Anja Steudle^1^, Dirk Spann^1^, Eva Pross^1^, Sri Karthika Shanmugam^2^, Ross E. Dalbey^2^ and Andreas Kuhn^1*^

Supplementary Information


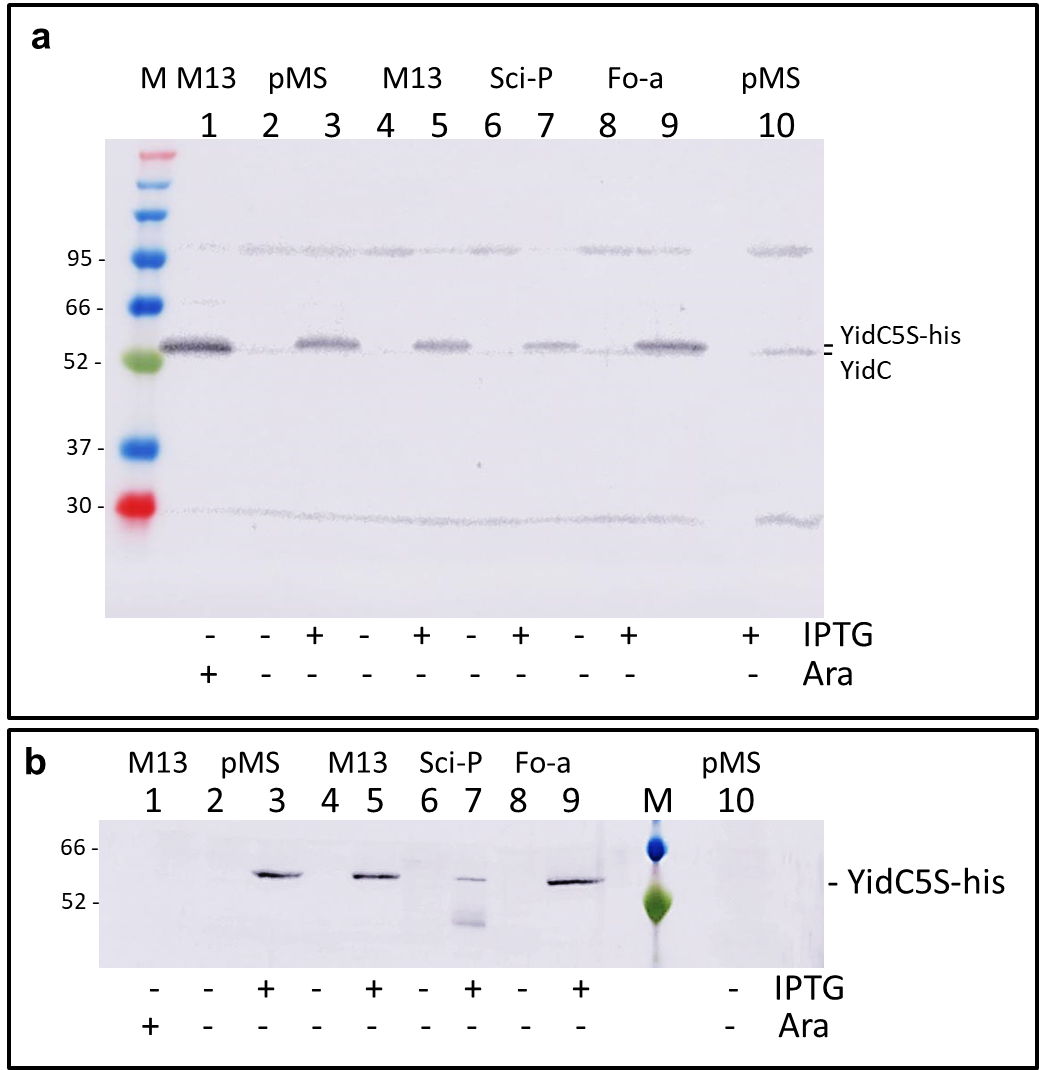


**Figure S1. Depletion of endogenous YidC and expression of YidC-5S.** (a) MK6 cells were grown under the same conditions Figure 2. In the absence of arabinose and IPTG the endogenous YidC expression was depleted. In presence of IPTG the YidC-5S mutant was expressed and detected on a Western blot with a YidC antiserum. (b) As in a, but YidC-5S was detected with an anti-his antibody to show the expression of YidC-5S under the different conditions.


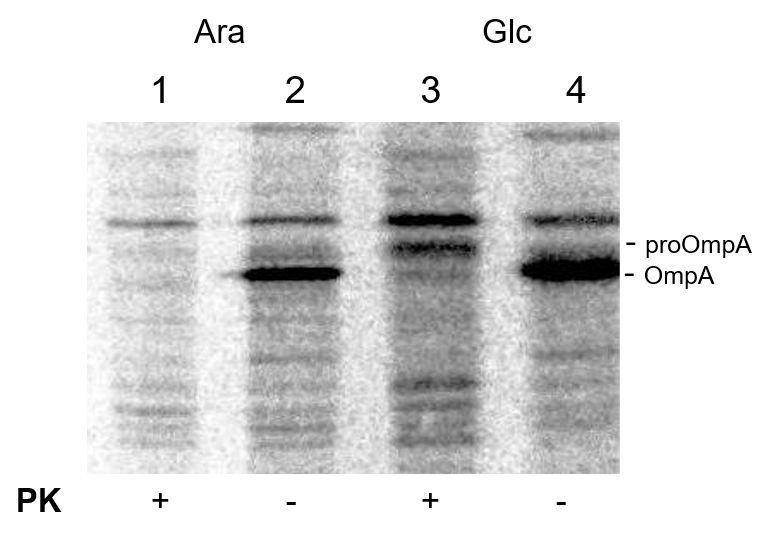


**Figure S2. Protease mapping control.** The protease activity and the cellular integrity was analysed by the proOmpA and OmpA content. An aliquot of the experiment shown in Fig. 2C was taken to evaluate proOmpA and OmpA after immunoprecipitation. Whereas proOmpA was protected from the proteinase K (PK, lanes 1, 3), the translocated mature OmpA protein was digested (lower band).


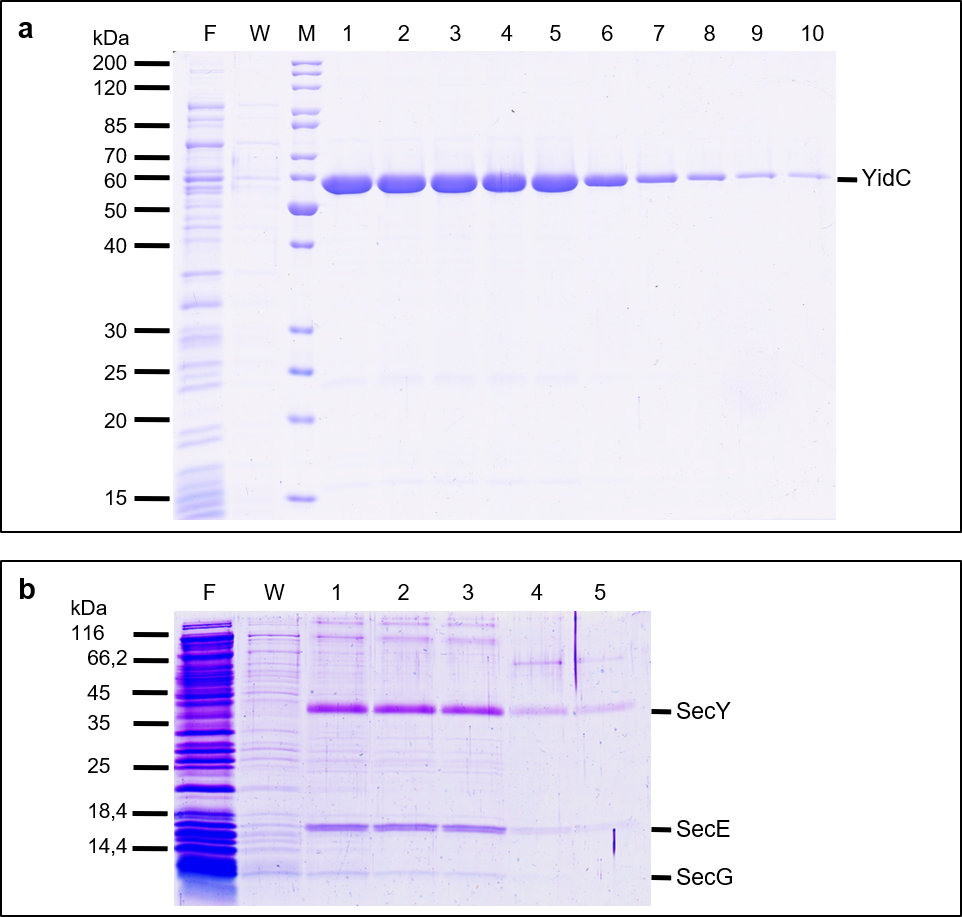


**Figure S3. Purification of YidC and SecYEG.** YidC (a) and SecYEG (b) were purified as described in the Methods section. Shown are the fractions of the IMAC, F=flow through, W=wash fraction, the numbers refer to the elution fractions.


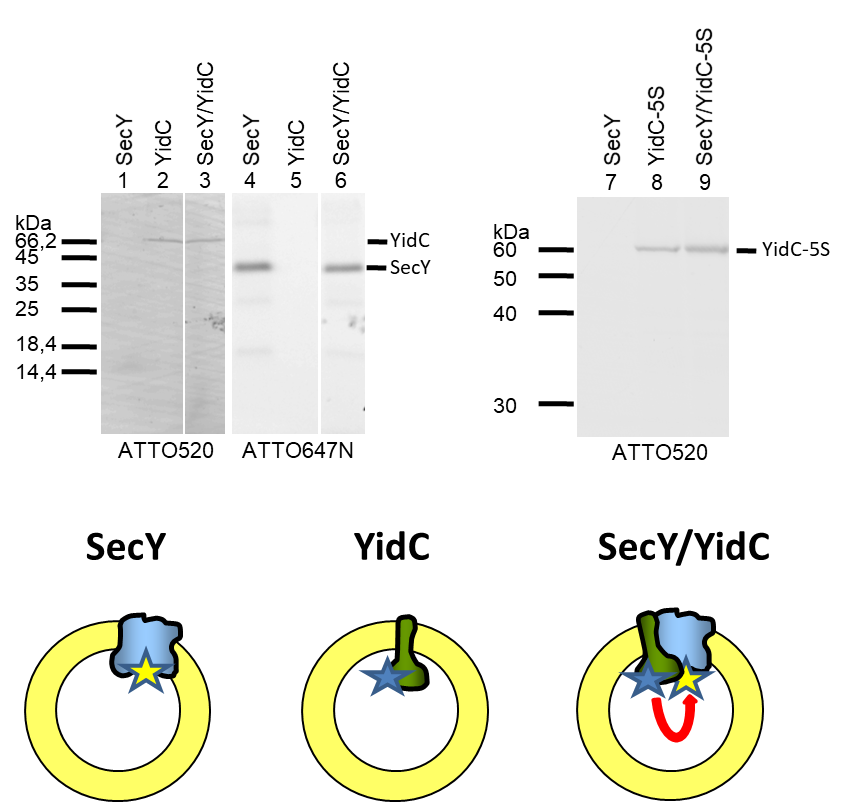


**Figure S4. FRET between SecYEG and YidC** **in proteoliposomes.** SecYEG-Atto647N (lanes 1, 4, 7) and YidC-Atto520 (lanes 2, 5) or YidC 5S-Atto520 (lane 8) were reconstituted separately into liposomes or co-reconstituted (lanes 3, 6, 9). After FRET measurements, the samples were analysed by SDS-PAGE and fluorescence imaging to corroborate the equal amounts of labelled proteins in the samples.


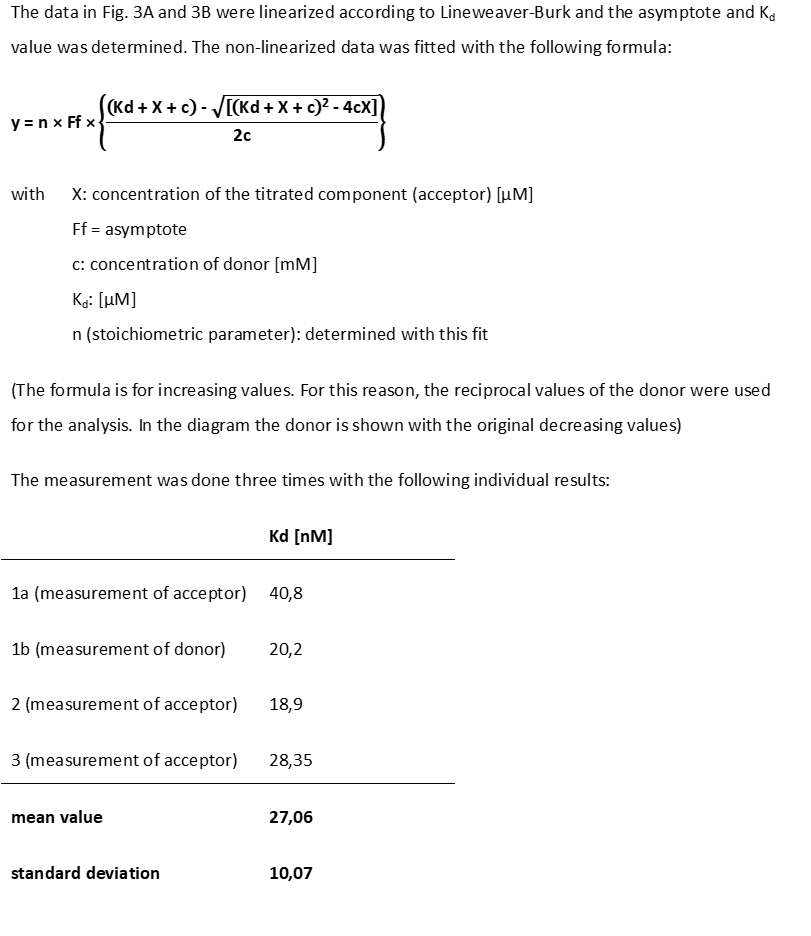


**Figure S5. Data processing and reproducibility of the FRET binding experiment.** The data of Fig. 3a and 3b were fitted as described here. The experiment was repeated three times with comparable results.


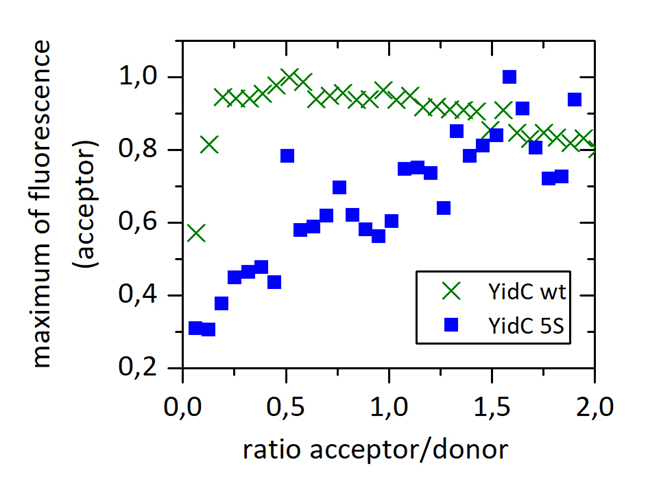


**Figure S6. Comparison of YidC wt and YidC-5S regarding the SecY/YidC-binding.** The FRET binding experiment in Fig. 3 was also done with the YidC-5S mutant in 0.03% dodecyl maltoside. Whereas there is an early saturation in case of the wildtype YidC (green crosses), the mutant (blue boxes) shows a non-specific binding.


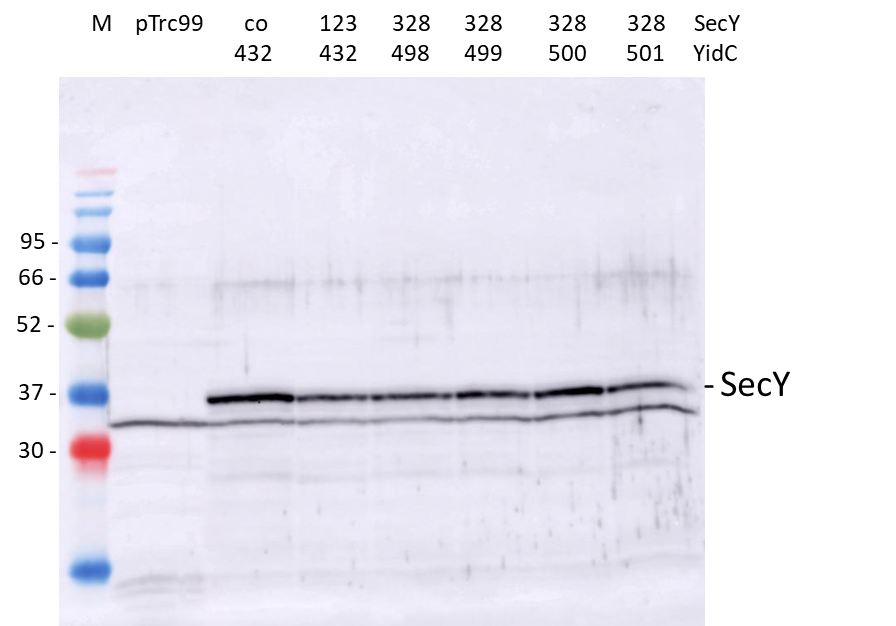


**Figure S7. Expression of SecY.** The expression for the different SecY mutants in combination with the different YidC mutants of the cross-linking study was shown on a Western blot with an anti-his antibody.

**Fig. 2a Fig.2b Fig.2c**


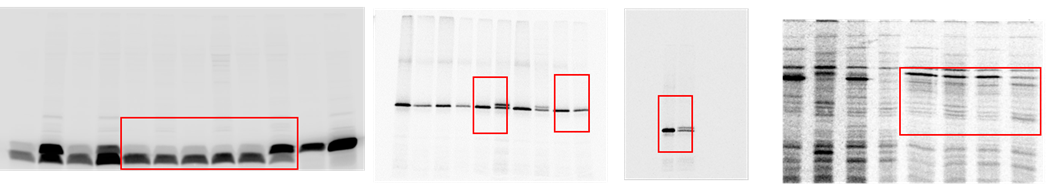
 Lanes 1-6 Lanes 1,2 5,6 3,4 Lanes 1-4

**Fig. 5,** Lanes 1-4 5-12 **Fig. S1b,** Lanes 1-10 **Fig.S2**, Lanes 1-4

**
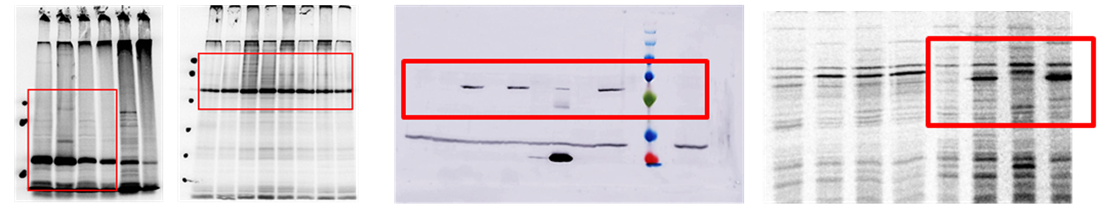
**

**Fig. S4**


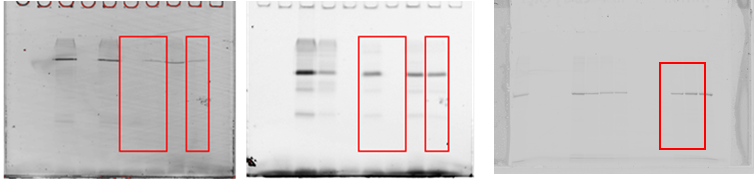


Lanes 1,2 3 4, 5 6 7-9

**Figure S8. Full-length gels of Figure 2, 5, S1, S2 and S4.**
